# Molecular prediction of lytic *vs* lysogenic states for *Microcystis* phage: Metatranscriptomic evidence of lysogeny during large bloom events

**DOI:** 10.1371/journal.pone.0184146

**Published:** 2017-09-05

**Authors:** Joshua M. A. Stough, Xiangming Tang, Lauren E. Krausfeldt, Morgan M. Steffen, Guang Gao, Gregory L. Boyer, Steven W. Wilhelm

**Affiliations:** 1 Department of Microbiology, University of Tennessee, Knoxville, TN, United States of America; 2 State Key Laboratory of Lake Science and Environment, Nanjing Institute of Geography & Limnology, Chinese Academy of Sciences, Nanjing, PR China; 3 Department of Biology, James Madison University, Harrisonburg, VA, United States of America; 4 College of Environmental Science and Forestry, The State University of New York, Syracuse, NY, United States of America; Laval University, CANADA

## Abstract

*Microcystis aeruginosa* is a freshwater bloom-forming cyanobacterium capable of producing the potent hepatotoxin, microcystin. Despite increased interest in this organism, little is known about the viruses that infect it and drive nutrient mobilization and transfer of genetic material between organisms. The genomic complement of sequenced phage suggests these viruses are capable of integrating into the host genome, though this activity has not been observed in the laboratory. While analyzing RNA-sequence data obtained from *Microcystis* blooms in Lake Tai (*Taihu*, China), we observed that a series of lysogeny-associated genes were highly expressed when genes involved in lytic infection were down-regulated. This pattern was consistent, though not always statistically significant, across multiple spatial and temporally distinct samples. For example, samples from Lake Tai (2014) showed a predominance of lytic virus activity from late July through October, while genes associated with lysogeny were strongly expressed in the early months (June–July) and toward the end of bloom season (October). Analyses of whole phage genome expression shows that transcription patterns are shared across sampling locations and that genes consistently clustered by co-expression into lytic and lysogenic groups. Expression of lytic-cycle associated genes was positively correlated to total dissolved nitrogen, ammonium concentration, and salinity. Lysogeny-associated gene expression was positively correlated with pH and total dissolved phosphorous. Our results suggest that lysogeny may be prevalent in *Microcystis* blooms and support the hypothesis that environmental conditions drive switching between temperate and lytic life cycles during bloom proliferation.

## Introduction

Viruses are one of the most potent drivers of nutrient cycles, horizontal gene transfer, and microbial evolution in aquatic ecosystems [1, 2]. Bacteriophage play an important role in microbial communities by lysing primary producers and heterotrophic bacteria, releasing nutrients from biomass [3]. Moreover, due to their density-dependent infection, viruses are thought to reduce the competitive advantages of some of the most prolific organisms–the “*kill-the-winner”* hypothesis [4]. Phage genomes also can encode auxiliary metabolic genes that serve to augment host metabolism during infection, considerably altering the functional potential of entire populations within the microbial community [5, 6]. Despite their recognized importance, much of the potential of viruses remains uncharacterized, highlighting a crucial need for examination of the role they play across ecosystems.

*Microcystis aeruginosa* has repeatedly been identified as a nuisance bloom-former in freshwater systems over the last several decades [7]. It has come to the forefront of public attention as the primary agent in blooms worldwide and for its ability to produce a potent hepatotoxin, originally known as “Fast-Death Factor” [8], but now known as microcystin [9, 10]. Recent impacts include the shutdown of the public water supply to the City of Toledo (Ohio) during the *Microcystis* bloom in 2014 [11], and the considerable accumulation of toxic algal biomass in Lake Tai, China (*Taihu* in Chinese) [12, 13]. While significant strides have been made describing the ecology [14–16], physiology [17–19], and genetics [20–22] of *Microcystis*, little is known about the effect of phage on *Microcystis* ecology. To date, only 11 viruses infecting *M*. *aeruginosa* have ever been brought into culture [23–28], of which only 2 have sequenced genomes [29, 30], and each of these isolates has subsequently been lost to science. *Microcystis* phage Ma-LMM01, classified as an unassigned myovirus, has been the best studied. The availability of Ma-LMM01’s full genome sequence has led to analyses of distribution (*via* PCR and qPCR-based techniques) and some characterization of its genetic regulation [31, 32].

Ma-LMM01 appears to have been host specific in lab studies, targeting *M*. *aeruginosa* at the strain level [27]. This has led to the hypothesis that phage play a role in modulating dominant strains during blooms [33]. Ecologically, one gene from this virus (*gp91*), encoding a viral tail sheath and present in the genomes of both *Microcystis* phages Ma-LMM01 and MaMV-DC [29, 30], has been used *via* qPCR to suggest *Microcystis*-specific phage particles can be present at concentrations >10,000 mL^-1^ of lake water [31, 34]. These virus densities and a projected high level of host specificity suggest the potential for long-term predator-prey coevolution between virus and host, a trait generally associated with temperate phage [35]. They also suggest that bloom events of susceptible *Microcystis* cells should quickly succumb to phage infection [4].

Beyond an ability to infect and lyse *Microcystis*, the Ma-LMM01 genome encodes machinery necessary for lysogeny and induction, including 3 transposases, a serine recombinase, and 2 prophage anti-repressors. In addition, one transposase (*gp135*) and the recombinase (*gp136*) make up a 2-gene mobile genetic element called IS607, originally identified in *Helicobacter pylori*, and has led some to hypothesize that these genes further act independently as a transposon [36, 37]. Although there is an absence of lysogenic activity with *Microcystis* observed in the laboratory, expression of these genes has been documented in environmental samples (although they were not tied to lysogeny [38]). Taken together, the presence of lysogeny-associated genes within *Microcystis* and the implied protection against superinfection might explain how this genus can come to dominate freshwater ecosystems and escape Hutchinson’s *Paradox of the Plankton* [39] or the “*kill-the-winner”* phenomenon [4].

During analyses of metatranscriptomic data from *Microcystis* blooms in Lake Tai, we observed expression of phage-encoded lysogeny-associated genes that negatively correlated with expression of genes consistent with lytic infection and phage replication. Regulation of these putative lysogenic genes appears to be strongly associated with specific environmental conditions in the water column. Based on these observations, we hypothesize that a phage lysogenizes the *Microcystis* bloom community in a matter constrained by nitrogen and phosphorus availability.

## Materials and methods

### Sample collection and survey of environmental conditions

No specific permissions were required for these locations/activities as Lake Tai is open access water. Research did not involve endangered or protected species. Samples were obtained from Lake Tai over the course of five months during the *M*. *aeruginosa* bloom in 2014 and have been used in conjunction with several other experiments [12]. Surface water samples were collected monthly from June to October from 11 different locations across the lake (S1 Table). From all stations and dates 35 samples were selected (based on the quality and quantity of extracted RNA) were submitted for RNA-seq. Samples from Lake Tai (25–180 mLs) were collected on 0.2-μm nominal pore-size Sterivex™ (EMD Millipore Corporation, Darmstadt, Germany) and preserved for transport by adding ~ 2 mL of RNA*later* (ThermoFisher Scientific, Waltham, MA).

Water column depth and Secchi depth (SD) were measured using a water depth gauge (Uwitec, Austria) and Secchi disk, respectively. Water temperature, electrical conductivity (EC), pH, dissolved oxygen (DO) and phycocyanobilin (PC) were measured *in situ* using a multiparameter water quality sonde (YSI 6600 V2, Yellow Springs Instruments Inc., USA). Total nitrogen (TN), total dissolved nitrogen (TDN), ammonium (NH_4_), nitrate (NO_3_), total phosphorus (TP), total dissolved phosphorus (TDP), orthophosphate (PO_4_), total dissolved solids (TDS), and chlorophyll *a* (chl *a*) were all measured according to standard methods [40].

Cyanobacterial toxins were determined using liquid chromatography coupled with mass spectroscopy as previously described [41]. Fourteen common microcystin congeners were determined by reverse phase liquid chromatography (microcystins RR, dRR, mRR, hYR, YR, LR, mLR, dLR, AR, FR, LA, LW, LF, WR and R-NOD) using a Waters ZQ4000 mass spectrometer coupled with a photodiode array spectrometer. Microcystins were all quantified against a microcystin-LR standard, and their presence confirmed using diagnostic ADDA UV signatures. We also looked for anatoxin-a (ATX), homoanatoxin-a, cylindrospermopsin (CYL) and deoxycylindrospermopsin in these extracts using HPLC coupled with mass selective (LCMS) or tandom mass (LC-MS/MS: Waters TQD) detection, and quantified against respective standards. Method detection limits were dependent on the volume filtered, ranging from 0.1–0.3 μg MC-LR / L and were less than 0.01 μg/L for anatoxin-a, cylindrospermopsin, and their variants.

### RNA extraction and sequencing

Total RNA was extracted using the MOBIO PowerWater (now Qiagen DNeasy PowerWater) DNA isolation kit for Sterivex (Qiagen, San Diego, CA) modified and optimized for RNA isolation. RNA concentration and purity were determined using a NanoDrop™ ND-1000 spectrophotometer. Extracted RNA was tested for DNA contamination by running a polymerase chain reaction using universal bacterial 16S rDNA primers 27F and 1522R (sensitivity ~ 10 gene copies per sample). The On-Spin Column DNase I Kit (MO BIO Laboratories) was used for DNA removal, with the modification that DNase was allowed to sit for up to 30 min to increase the efficiency of DNA removal. Purified RNA samples were shipped to the Hudson Alpha Institute Genomic Services Laboratory (Huntsville, AL) for rRNA reduction, using the Ribo-Zero Gold Epidemiology rRNA removal kit, and sequencing on the Illumina HiSeq™ platform using a paired-end 125 bp flow cell.

### RNA-seq data processing

Raw sequences were processed using the CLC Genomics Workbench v. 9.5.4 suite (QIAGEN, Hilden, Germany). Bases below 0.03 error score cutoff were trimmed. Samples were subjected to a subsequent *in silico* rRNA reduction using the SortmeRNA 2.0 software package [42]. Filtered paired-reads were competitively mapped to cyanobacterial and phage genomes (S2) with a 0.9 read-length fraction and 0.9 identity-fraction cutoffs. Transcripts were enumerated as read pairs mapped within the open reading frames of individual genes, and counts normalized by library size (unless noted). Paired reads with ends mapping to different genomes were not included in downstream analyses or counts. Sequence information has been deposited in MG-RAST database under the study Lake_Taihu_metatranscriptome_project (sample IDs in S1 Table).

### Phylogenetic analysis

Reference sequences from Proteobacteria, Cyanobacteria, and phage identified by sequence alignment as IS607 regions in [36] were downloaded from NCBI (S3 Table). IS607 reference sequences were aligned in MEGA 7.0.14 software [43] using the MUSCLE algorithm [44] and this alignment was then used to generate a maximum likelihood tree with a Shimodaira-Hasegawa-like approximate likelihood ratio test branch validation using PhyML [45]. The reference sequences were then aligned with RNA-seq reads mapping to the Ma-LMM01 IS607 region in HMMER v. 3.1 (hmmer.org). Reads from the alignment were placed the reference tree using pplacer [46]. Quantity of reads placed on the tree was visualized as branch width using the guppy software package [47].

### Statistical analysis

*Microcystis* phage Ma-LMM01 gene read counts were log_2_(x+10) transformed and Pearson correlation values were calculated in R Statistics [48] using the Hmisc R package [49]. Mapped read counts per gene were normalized to expression of *M*. *aeruginosa* NIES-843 *rpoB* (as a proxy for host cell density) and plotted using the SigmaPlot software package (Systat Software, Chicago, IL). Whole genome expression was determined by counting reads mapped within gene regions on the Ma-LMM01 reference genome, which were normalized by library size, square root transformed, and used to generate a Bray-Curtis dissimilarity matrix and non-metric multidimensional scaling (nMDS) plots in the PRIMER7 software suite [50]. Associated environmental variables were correlated with Bray-Curtis dissimilarity distributions and plotted as vectors on the nMDS. The relationship between environmental variables and expression of the phage genome was determined using the BEST analysis [50]. The co-occurrence of expression of whole genome expression was grouped using the CLUSTER function using the Pearson correlation coefficient as the index of association with a 0.1 p-value cutoff. The results of this analysis were visualized in a dendrogram, all in PRIMER7.

## Results

### Differential expression of genes from *Microcystis*-infecting phage

**Fig 1:**
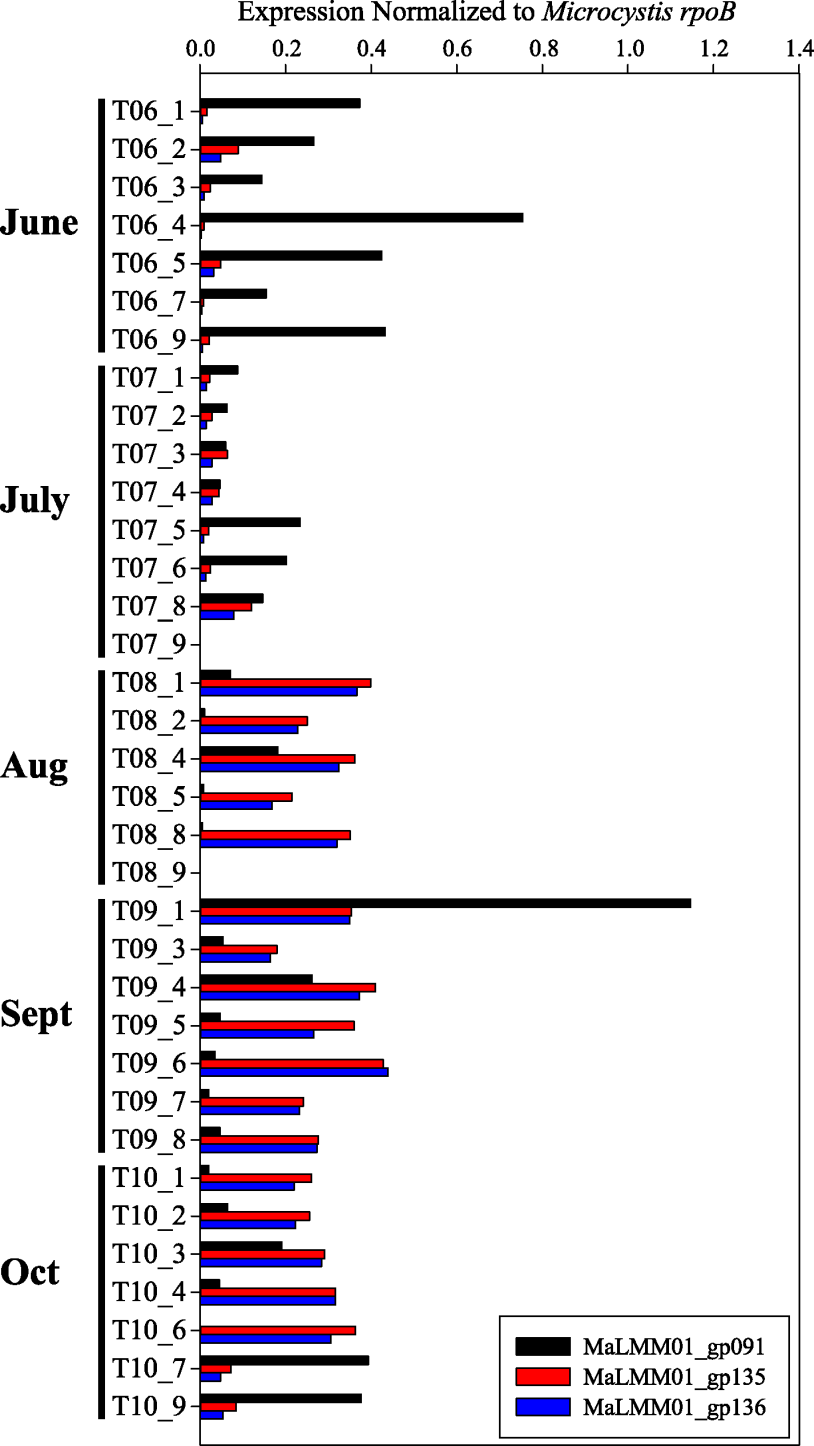
Lytic and lysogenic gene expression by station. Spatial and temporal gene expression of lytic and lysogenic genes from *Microcystis*-phage in Lake Tai. Expression of the *Microcystis* phage Ma-LMM01 phage viral tail sheath (*gp091*, black), transposase (*gp135*, red), and recombinase (*gp136*, blue) normalized by expression of *Microcystis aeruginosa* RNA polymerase B (*rpoB*) observed in the Lake Tai dataset.

**Fig 2:**
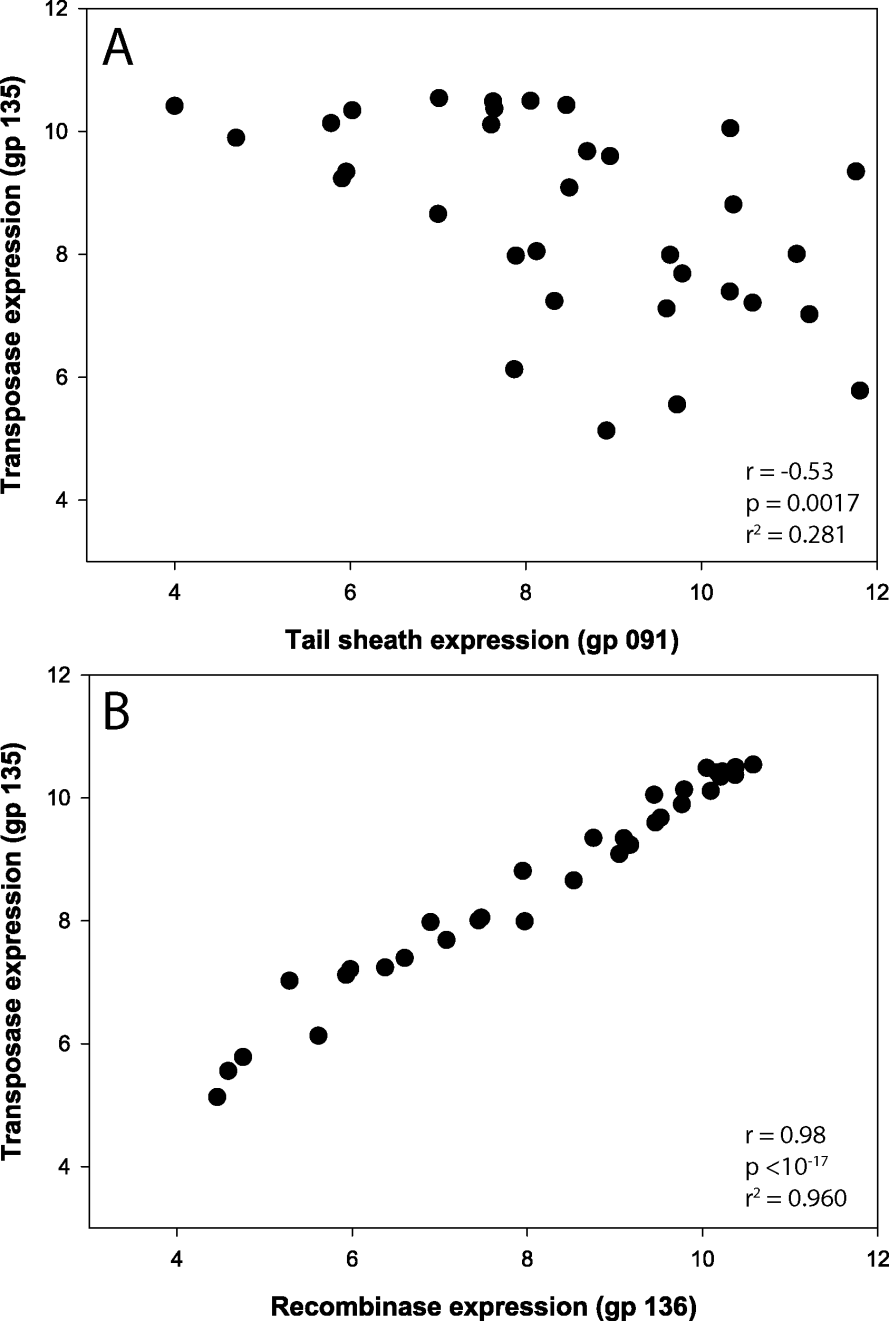
Tail sheath, transposase, and recombinase coexpression. Co-expression of genes associated with putative lytic and lysogenic infections in Lake Tai. A. Scatterplot comparing expression of Ma-LMM01 viral tail sheath (*gp091*, *x*-axis) to viral transposase (*gp135*, *y*-axis). Expression values are absolute read abundance log_2_ normalized and demonstrate the negative relationship between the putative lytic (*gp091*) and lysogenic (*gp135*) infection markers. B. Scatterplot comparing expression of Ma-LMM01 recombinase gene (*gp136*, x-axis) to viral transposase (*gp135*, y-axis), both putative markers of lysogenic infection of *Microcystis*.

Normalized expression of the Ma-LMM01-like tail sheath (*gp091*), transposase (*gp135*), and site-specific recombinase (*gp136*) observed in Lake Tai are shown in Fig 1. Of the 35 samples, 2 (T07_9 and T08_9) exhibited negligible expression of phage and host genes and have been removed from subsequent analyses. In the remaining 33 samples, 16 showed more abundant expression of *gp091* relative to *gp135*, with a ratio ranging from 1.21 to 79-fold, implying that lytic infection was dominant. These samples were collected during the earlier months (June and July) of the bloom season, with the exception of T09_1, T10_7, and T10_9, which were collected during September and October. The remaining samples showed expression of the *gp135* and *gp136* to be greater than the expression of *gp091*, implying the *Microcystis* community was, at least to some degree, lysogenized. These samples primarily occurred during the months of August, September, and October. Statistically, sample location within the lake did not relate to expression patterns, with each station exhibiting periods with dominance of lytic or putative-lysogenic transcripts almost in equal measure across all five months. Tail sheath expression was significantly and negatively correlated with both transposase (Fig 2, ρ = -0.53, *p* = 0.0017) and also recombinase abundance (ρ = -0.57, *p* = 0.0001). Transposase and recombinase were very highly correlated (ρ = 0.98, *p* = 0, R = 0.986 on a linear function fit), suggesting tightly coordinated co-expression.

### *Microcystis*-phage genome expression

**Fig 3:**
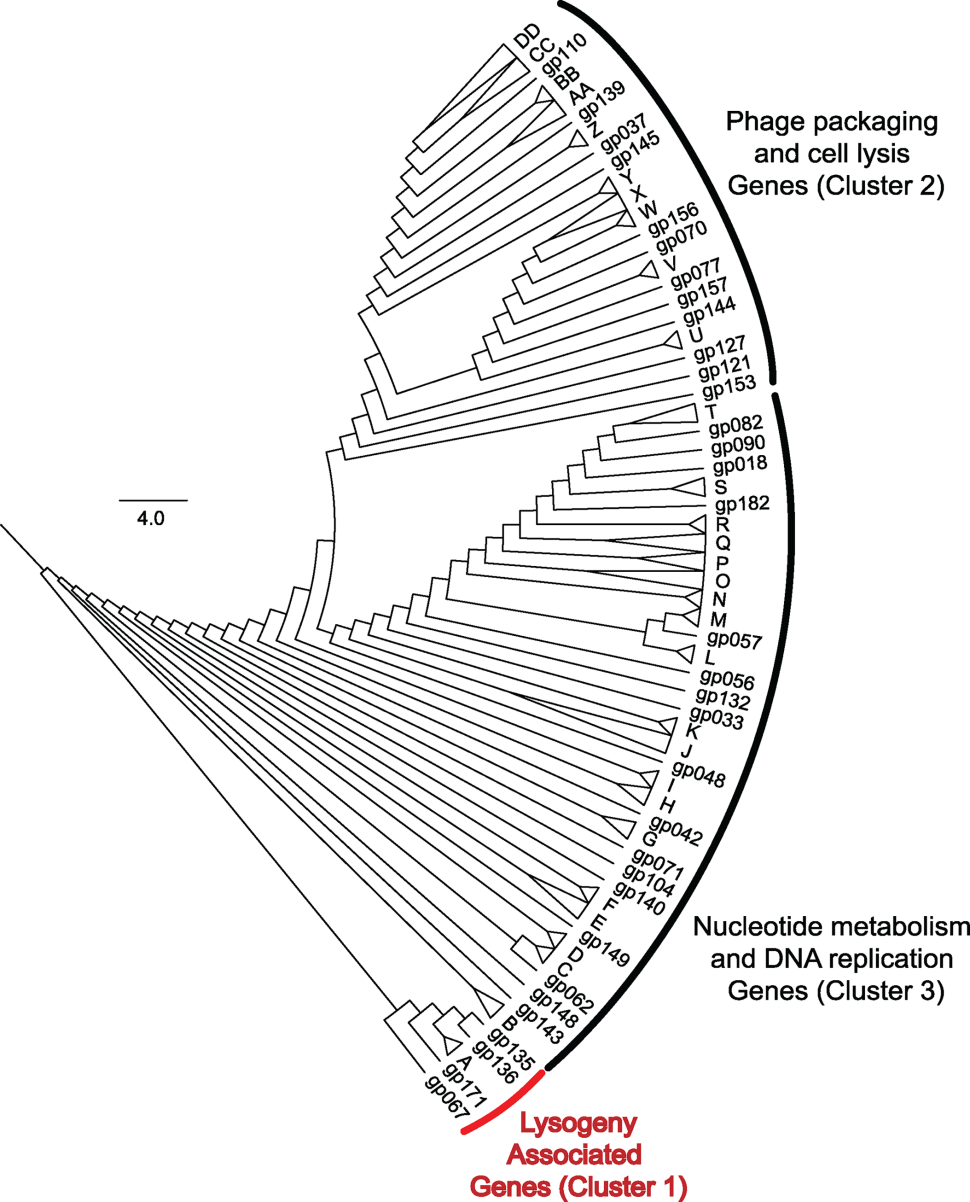
Ma-LMM01 whole genome coexpression. Cluster analysis of statistically co-expressed *Microcystis*-phage gene expression (based on Ma-LMM01 genome) in Lake Tai. Individual branches represent genes correlated with the expression of others. Transcript sets are collapsed and labeled with a letter where expression patterns were statistically indistinguishable (see S4 Table for genes contained in collapsed branches).

As a proxy for *in situ* expression of all *Microcystis* phage genes, we recruited environmental transcripts to the Ma-LMM01 genome. Results observed from samples collected in Lake Tai, and organized by hierarchical clustering, are represented in Fig 3. Each of the genes for both datasets generally fell into one of three major clusters. The first cluster includes all the genes potentially involved in lysogeny, including all three transposases (*gp031* and *gp032*– collapsed in branch A, *gp135*), the serine recombinase (*gp136*), and two hypothetical proteins (*gp171*, *gp067*).

The second cluster is predominantly made up of genes involved in phage packaging and cell lysis. It contains 60 genes, including 2 encoding lysozymes (*gp069* –collapsed in branch W, and *gp095* –collapsed in branch X) and the genes for DNA terminase (*gp118*—collapsed in branch DD), DNA primase (*gp134*—collapsed in branch AA), and a putative Fe/S oxidoreductase (*gp128*—collapsed in branch AA), which are the only ORFs with functions assigned. These genes exhibit high correlation values (ρ ≥ 0.7), of which 48 are significantly co-expressed (p ≤ 0.1) with at least one other gene.

The third cluster is the largest, and is made up of genes whose products are associated with nucleotide metabolism, DNA replication, and the structural components of the phage. It is made up of 112 genes including the viral tail sheath (*gp091*, collapsed in branch T), phage-encoded RecA (collapsed in branch S), the phycobilisome degradation protein NblA (collapsed in branch N), and a rIIA-like protein (collapsed in branch P). Viral tail sheath expression was highly correlated with genes *gp088* and *gp092*, which were predicted by protein size to encode viral tail tube proteins. Genes *gp086* and *gp087* also clustered with the tail sheath, which are believed to encode major head proteins for the phage particle.

### Environmental drivers of phage gene expression

**Fig 4:**
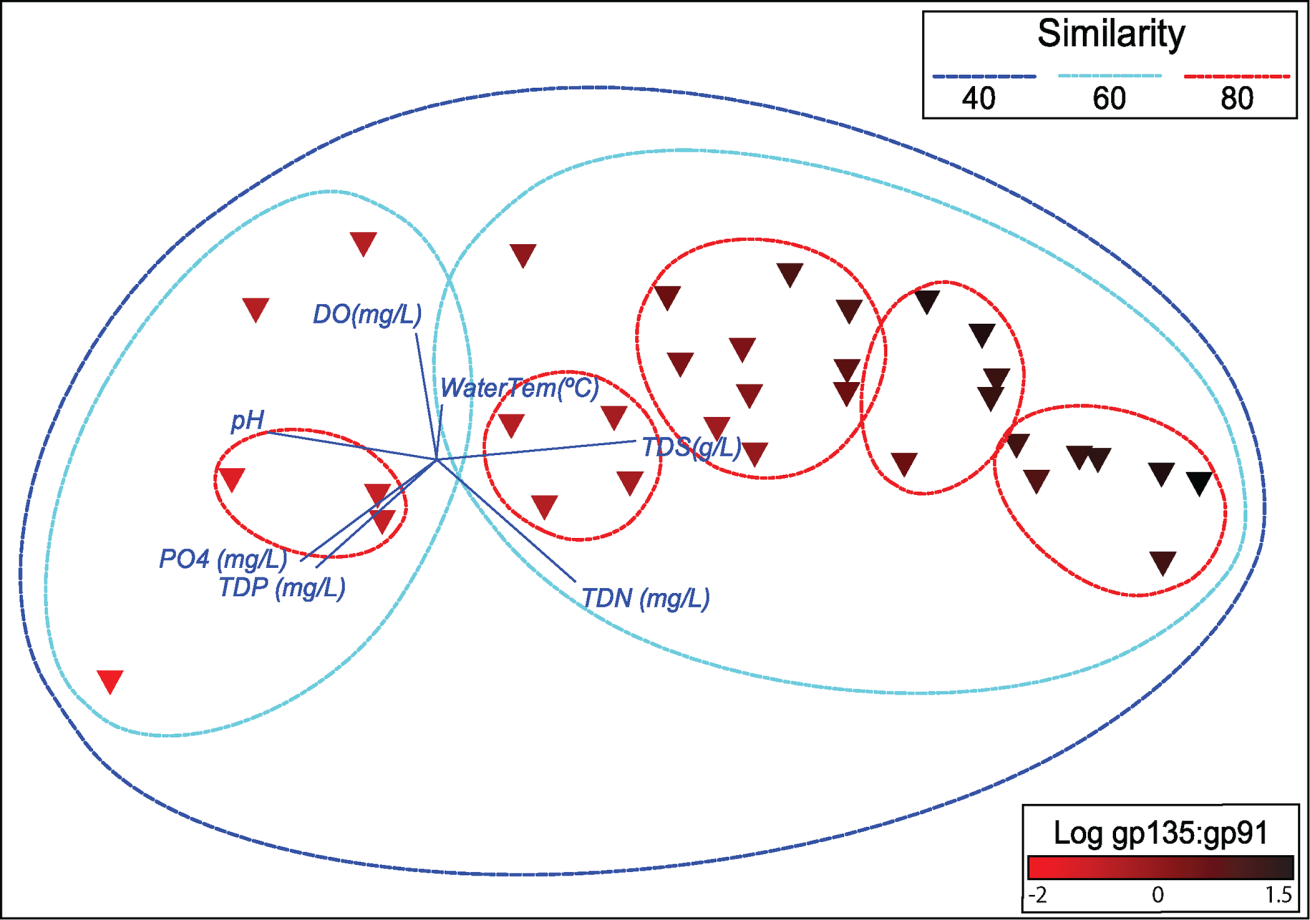
Environmental contribution to whole genome expression. A. Non-metric multidimensional scaling plot of Bray-Curtis dissimilarity between *Microcystis*-infecting phage whole genome expression for Lake Tai. Read abundance was normalized by library size and square root transformed. Ellipses represent minimum similarity between samples at the 40%, 60%, and 80% levels. Symbols have been colored based on the log_2_ transformed *gp091*:*gp135* expression ratio to denote lytic (black) vs lysogenic (red) dominated states. Environmental variables identified in the BEST analysis have been correlated (Pearson) with similarity between samples and plotted as vectors, indicating the direction on the 2-dimensional plane with which they correlated.

A non-metric multidimensional scaling (nMDS) plot of the Bray-Curtis dissimilarity analysis of phage genome expression in Lake Tai is shown in Fig 4. Samples were distributed in a continuum across the *x*-axis, forming two primary clusters where phage gene expression was at least 60% similar. The position of samples along the *x*-axis corresponds significantly to the ratio of tail sheath to transposon (*gp135*) expression (a similar trend was observed when the ratio of tail sheath to recombinase (*gp136*) expression is plotted on the samples). Vectors for environmental variables are plotted on the nMDS, showing that pH (towards lysogenic) and concentration of total dissolved solids (towards lytic) contributed most significantly to position along the x-axis. Total dissolved nitrogen and phosphorous also contributed to position along the x-axis, driving the position of samples towards greater expression of lytic genes or putative lysogenic genes, respectively. The dissolved oxygen concentration and water temperature also contributed, though more significantly to position along the *y*-axis. The BEST analysis of environmental variable contribution to expression of the entire phage genome confirmed these associations, and determined that water temperature, pH, and concentration of total dissolved solids, phosphorous, nitrogen, and oxygen concentrations were responsible for 33% of the variation in gene expression (*p* = 0.05). Phage gene expression was not correlated to toxin concentration (*gp091*: microcystin μg/L, ρ = -0.19, *p* = 0.2956; *gp135*: microcystin, ρ = 0.29, *p* = 0.099; *gp136*: microcystin, ρ = 0.25, *p* = 0.1623).

## Discussion

We surveyed community metatranscriptomes from natural populations of *M*. *aeruginosa* at “bloom densities” to describe the physiology and ecology of *Microcystis*, and in the process identified active phage infections by the *Microcystis* phage Ma-LMM01. We have analyzed this data in light of available nutrient concentrations, toxin levels, and environmental conditions to predict how lake chemistry and climate influenced *Microcystis* phage gene expression. Our observations suggest that expression across the entire phage genome appears to have switched between the expression of genes involved in active viral replication (*i*.*e*., the lytic cycle), and the expression of genes that have been proposed to allow the phage to integrate into the host genome (*i*.*e*., lysogeny). Lastly, we found that the expression of phage genes appears to have been strongly associated with total dissolved solids and pH as well as the availability of nutrients, specifically the relative abundance of nitrogen and phosphorous. These observations have given rise to three distinct hypotheses: 1.) These correlations and co-occurrences are the product of random chance; 2.) The pattern of gene expression represents a novel physiological interaction (the purpose of which is currently unclear) between this phage and its host and was independent of lysogeny; 3.) The results indicate that *Microcystis* phage were actively switching between lytic and lysogenic cycles. We address these conclusions below within the context of factors that drive freshwater microbial communities.

The possibility that observed patterns in phage gene expression were the result of random chance is not supported by our analyses. The observation of similar expression patterns across Lake Tai suggests the mechanism by which *Microcystis*-infecting phage regulate gene expression has been largely conserved and is important for this virus’s survival. Previous attempts to describe Ma-LMM01 transcriptional regulation in the laboratory relied on ORF orientation in the virus genome sequence, which yielded two general groups of genes: an “early” gene region containing 144 genes that were suggested to be responsible for nucleotide metabolism and genome replication, and a late gene region, encoding the remaining 40 genes, believed to encode phage structural components [29]. A subsequent study used q-rtPCR in culture to measure transcripts of the viral tail sheath (*gp091*), a putative late gene, and the gene for the phycobilisome degradation protein (NblA), a putative early region gene [32]. They observed a temporal separation of expression between the two genes and hypothesized that the larger gene regions were consistent with early/late phage gene expression, a regulation strategy observed in other cyanomyoviruses [51]. The disconnect between the regions identified by Yoshida and colleagues with our clustering is not surprising: in dealing with natural populations (unlike lab studies), we were most likely dealing with non-synchronous infections. Indeed, that there are statistically relevant relationships within the expression data suggests there are strong environmental controls on lytic *vs* lysogenic decisions.

**Fig 5:**
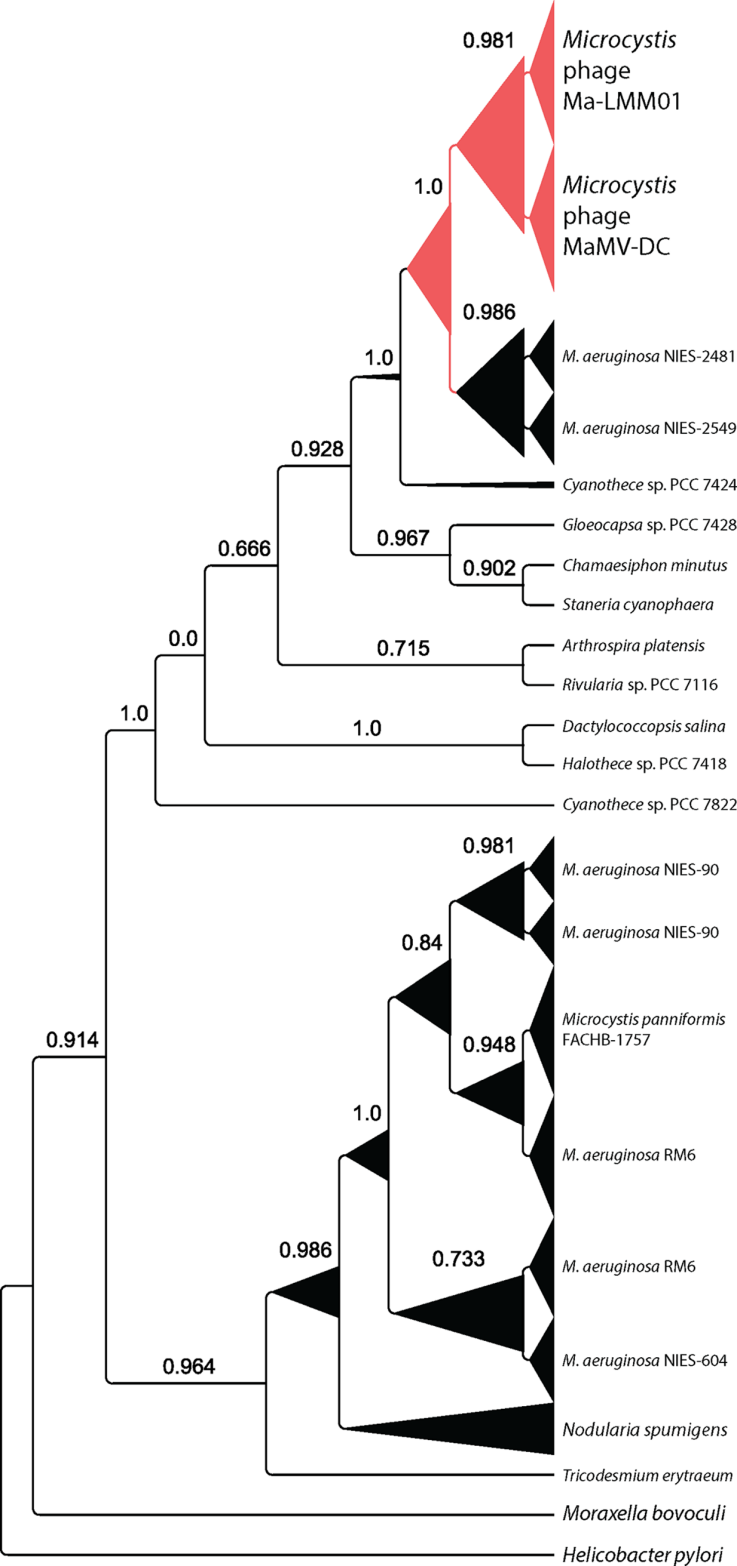
Phylogenetic distribution of IS607 reads. Bootstrapped phylogenetic tree of the mobile element IS607 where branch widths indicate abundance of Lake Tai dataset reads mapping to that branch. Branches belonging to the *Microcystis* phage are colored red.

A second potential explanation for our observations, that switching between expression states is unrelated to lysogeny, remains plausible. Much of the gene expression we attributed to genome integration originates in the virus’ three putative-transposases (*gp031*, *gp032*, *gp135*) and the recombinase (*gp136*), all of which have some homologues in different strains of *M*. *aeruginosa* and other cyanobacteria. Transposase *gp135* belongs to a potential family of mobile elements, IS607, which was originally identified in *Helicobacter pylori* [37]. IS607 representatives encode a corresponding serine recombinase (*gp136* in Ma-LMM01) and together, this gene pair is widespread amongst sequenced cyanobacteria [36]. While these genes can be phylogenetically resolved across the length of the insertion sequence, determining the genomic origin of short sequencing reads is more challenging. Our pplacer phylogenetic tree (Fig 5) demonstrates the majority of reads were identified as viral in origin, but the dearth of sequenced phage genomes related to Ma-LMM01 makes it difficult to evaluate the consistency of IS607 in viruses. Additionally, the IS607 encoded serine recombinase is atypical in structure amongst other similar enzymes. The DNA-binding and catalytic domains are flipped in orientation, resulting in a recombinase that acts *via* a modified mechanism, leading to a significant reduction in insertion site specificity [52]. At the outset, it is not known how this would influence activity of the insertion sequence in the context of viral infection, nor how it could play a role in lysogeny, but we speculate that decreased binding specificity might better allow integration of the virus into the notoriously plastic *M*. *aeruginosa* genome [20, 22]. It should also be noted that the presence of insertion sequences in phage genomes are very rare, as they can negatively impact virus survival [53].

That observed shifts in *Microcystis*-phage gene expression represent active genome integration (lysogeny) are the most consistent with our observations and those in other systems. Moreover, that this process is tied to nutrient availability in the water column gives this observation significant ecological relevance. The formation of a lysogen would explain why putative lysogenic genes are conserved in the phage genome in a variety of geographic locations [38]. There is a broad literature suggesting that phage have adapted to replicate or integrate depending on the conditions that favor the growth or senescence of their particular host [54–57]. Nutrient availability has long been associated with the formation of prophage in environmental systems, though it is generally thought to inhibit induction indirectly by limiting the material available to produce viral progeny, rather than by direct sensing for lysis-lysogeny decision making [58]. In better characterized phage-host systems, such as Lambda phage, the richness of the growth medium modulates signals in host metabolism that influence the lysis-lysogeny decision [59]. Unfortunately, our ability to determine the mechanism of action from metatranscriptomic data is limited, and the lack of similarity to better characterized phage systems, such as Lambda phage, makes comparisons with *Microcystis* phage difficult to draw at this time. This is further complicated by the current unavailability of *Microcystis*-infecting phage for controlled studies. That said, it is clear from the consensus of the scientific community that we cannot discount the importance of this (and similar) environmental molecular studies [60].

That *Microcystis* blooms can proliferate to massive densities [61] and yet somehow escape infection by the community of abundant phage [62] remains a perplexing ecological problem. This may be explained by the ability to resist infection by lytic viruses due to lysogen-induced resistance to superinfection. Indeed, while many observations lie in contrast, other studies that have suggested a “*Piggyback-the-winner*” model [63], which proposes that the spread of viral genomic material is best served by lysogenizing rapidly growing host cells that can persist at high densities. Clues to how this occurs mechanistically may lie in the uncharacterized genes co-expressed with the transposase and recombinase, namely *gp171* and *gp067*. While neither of these genes have close hits in the NCBI database, their implied relationship with the putative lysogenic genes suggests involvement in prophage maintenance. However, without culture work to identify their function, this remains speculation.

We observed that *Microcystis* phage gene expression could consistently be detected in *Microcystis* blooms and that a dramatic shift expression of lytic vs lysogenic gene groups was tied to environmental cues. Although the cause and effect of these cues needs further study, we hypothesize that *Microcystis*-infecting phage may actively integrate into the host genome–a state that can be distinguished from the lytic cycle *via* the relative transcription of *gp091* and *gp135*. While these new observations need continued validation and a better resolution of mechanistic controls, this study demonstrates that phage may have a strong influence population dynamics of this harmful bloom forming species.

## Supporting information

S1 Table Sampling information. Name, date, time, and location of each of the samples taken from Lake Tai and used for metranscriptomic sequencing. Also recorded is the environmental data collected for each sample, including water temperature during sampling (WaterTem °C), electric conductivity (EC), concentration of total dissolved solids (TDS), salinity (Sal), pH, nephelometric turbidity unit (NTU), YSI chlorphyll (YSI-CHL), phycocyanin (PC), dissolved oxygen (DO), Secchi depth (SD), depth of the lake at the sampling site (WaterDep), total nitrogen concentration (TN), total dissolved nitrogen concentration (TDN), ammonium concentration (NH4), total phosphorous concentration (TP), total dissolved phosphorous (TDP), phosphate concentration (PO4), and chlorophyll A concentration (CHLa).
(XLSX)

S2 Table Read mapping statistics. Statistics from mapping metatranscriptomic reads to reference genomes from each of the Lake Tai samples. Cells show the total number of reads mapped to each of the given genomes during competitive read mapping with a 0.9 similarity cutoff and a 0.9 length fraction cutoff.
(XLSX)

S3 Table Reference sequence information. Reference sequences used in Fig 5 and their accession numbers.
(XLSX)

S4 Table Gene branches collapsed in cluster diagram. Branches collapsed in Fig 3. Each of the collapsed branches is identified by the letter from Fig 3, and beneath are listed the genes whose expression patterns were statistically indistinguishable by hierarchical clustering.
(XLSX)
